# P-2351. Assumptions and Realities: Unraveling Varicella Zoster Virus Immunity in Unvaccinated Populations

**DOI:** 10.1093/ofid/ofae631.2503

**Published:** 2025-01-29

**Authors:** Sofia Jordão, Eduarda Pena, Maria João Gonçalves, Clara Batista, Susana Ramos Oliveira, Constança Azeredo, Sara Araújo, Fábio Reis, Cristina Soeiro, Ricardo Correia de Abreu

**Affiliations:** Hospital Pedro Hispano - Unidade Local Saúde Matosinhos, Matosinhos, Porto, Portugal; Hospital Pedro Hispano, Porto, Porto, Portugal; Hospital Pedro Hispano, Porto, Porto, Portugal; Unidade Local de Saúde de Matosinhos - Hospital Pedro Hispano, Porto, Porto, Portugal; Unidade Local de Saúde de Matosinhos - Hospital Pedro Hispano, Porto, Porto, Portugal; Unidade Local de Saúde de Matosinhos - Hospital Pedro Hispano, Porto, Porto, Portugal; Unidade Local de Saúde de Matosinhos - Hospital Pedro Hispano, Porto, Porto, Portugal; Hospital Pedro Hispano, Porto, Porto, Portugal; Unidade Local de Saúde de Matosinhos - Hospital Pedro Hispano, Porto, Porto, Portugal; Hospital Pedro Hispano - Unidade Local de Saúde de Matosinhos, Matosinhos, Porto, Portugal

## Abstract

**Background:**

In countries without universal vaccination for chickenpox in childhood, a very high seroprevalence for varicella zoster virus (VZV) IgG in adults is often assumed. Consequently, some suggest vaccinating at-risk adults against herpes zoster, even if they lack a documented history of chickenpox, under the assumption of immunity. However, this presumption may not accurately reflect today's epidemiological landscape. Neglecting the risk of primary infection post-childhood could lead to severe outcomes in vulnerable adults, pregnant patients, and increase the likelihood of exposure for caregivers, potentially resulting in secondary transmission.

**Figure:**
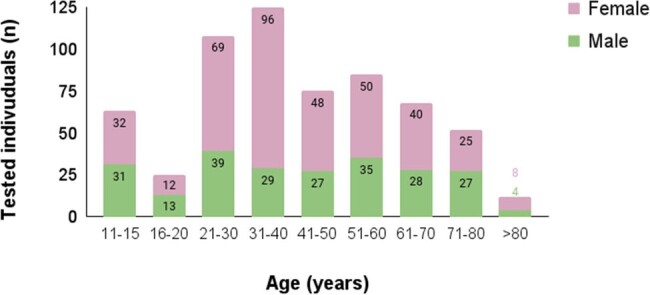

Graphic 1. Sample distribution by gender and age group.

**Methods:**

A retrospective study was conducted on VZV serology among individuals aged 11 and above, from February 2014 to March 2024, who were unvaccinated and lacked known chickenpox history, within a Portuguese hospital.

**Figure:**
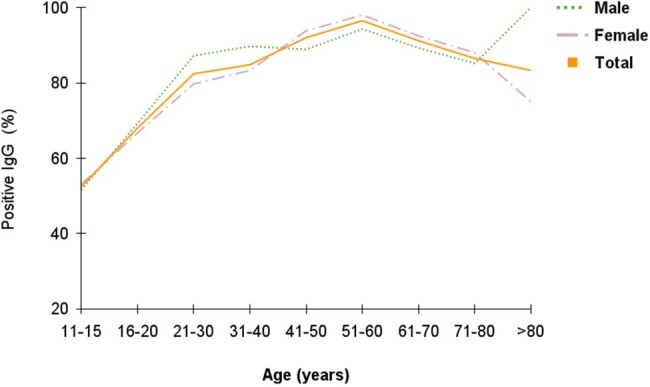

Graphic 2. Varicella Zoster Virus IgG seroprevalence by gender and age group.

**Results:**

Analysis of 613 patients (68% women, median age 39 years), all with high VZV infection risk due to comorbidities or immunosuppression, revealed an 83.6% VZV IgG positivity rate, increasing with age. Among working age individuals aged 21-60,12% were seronegative for VZV and 19.2% of women of childbearing age 16-40 showed no immunity.

**Conclusion:**

Although common, the presence of antibodies against VZV is not universal after childhood in unvaccinated populations. Vaccination for herpes zoster is not indicated for the prevention of primary infection, so assuming immunity may fail to prevent it in a population at risk. Prevention of chickenpox relies on specific vaccination, or the use of immunoglobulin when the former is contraindicated, which implies recognition of this susceptibility. Serology remains a useful tool, especially for at-risk individuals and caregivers, and should be considered in women of reproductive age and immunocompromised individuals.

**Disclosures:**

All Authors: No reported disclosures

